# What Determines Support for Donor Registration Systems? The Influence of Sociopolitical Viewpoint, Attitudes Toward Organ Donation, and Patients’ Need

**DOI:** 10.1007/s12529-019-09777-4

**Published:** 2019-02-28

**Authors:** Anton J. M. Dijker, Erica de Bakker, Stanneke C. Bensen, Nanne K. de Vries

**Affiliations:** Faculty of Health, Medicine and Life Sciences, Department of Health Promotion, CAPHRI, Maastricht University, P.O. Box 616, 6200MD Maastricht, The Netherlands

**Keywords:** Posthumous organ donation, Donor registration system, Opt-in versus opt-out, Sociopolitical viewpoints, Autonomy, Coercion, Reciprocity, Beliefs, Attitudes

## Abstract

**Background:**

In forming opinions about donor registration systems such as opt-in versus opt-out, the sociopolitical implications of these systems may be confounded with attitudes toward organ donation itself, causing people to talk at cross purposes. The goal of the present research was to examine the interactive effects of sociopolitical viewpoint, attitude toward donation (as evidenced by current registration status in study 1 and registration intention of unregistered individuals in study 2), and patients’ need for organs on people’s support for a particular system.

**Method:**

In study 1, we randomly assigned registered donors, registered nondonors, and nonregistered individuals to one of three sociopolitically inspired solutions to reducing the organ shortage, distinguishing between solutions based on autonomy, coercion by the state, and reciprocity, respectively. In study 2, we concentrated specifically on young and unregistered people in order to examine how prior donation intentions or indecision with respect to donor registration affect responses to the three different sociopolitical viewpoints. In both studies, we also manipulated salience of patients’ need.

**Results:**

Registered donors in study 1 and unregistered individuals with donation intention in study 2 (high in sympathy, low in anxiety) were highly and equally supportive of a solution based on autonomy and coercion. In contrast, registered nondonors in study 1 and unregistered and undecided individuals in study 2 (lower in sympathy, higher in anxiety) were less supportive of a solution based on coercion than autonomy. Study 2 also found that, for undecided individuals, a more salient need state was associated with a drop in anxiety and stronger support for coercion. Results for a system based on reciprocity were more difficult to interpret.

**Conclusion:**

Individuals most concerned with the need of patients waiting for an organ are relatively indifferent with respect to the sociopolitical implications of a registration system, while those strongly objecting to a coercive role for the state express reservations against organ donation itself. In order to help people to form balanced opinions about organ donation systems, we recommend to make the prosocial and sociopolitical aspects equally salient and deserving of debate.

## Introduction

Most countries employ one of two systems to solicit and register people’s choice with respect to donating organs after death. Countries may either use an opt-in system (people are considered nondonor by default but may give explicit consent to donate or “opt-in”) or opt-out system (presumed consent—people are considered donor by default but are allowed to object or “opt-out”). However, countries employing an opt-out system differ considerably in how objections to posthumous donation are obtained and registered. For example, in Belgium, people are required to personally visit the city hall in order to file an objection against posthumous donation and Spain even lacks an official system to register objections. Contrast this with the situation in the Netherlands that currently employs an opt-in system, but voted early this year in favor of an opt-out system according to which citizens are first provided with three opportunities to object against donation (in addition to register as a donor) before treating those still failing to register a choice as “not objecting” to donation.

Although the new Dutch system strongly encourages people to make an autonomous choice and therefore bears resemblance to an opt-in system (it is formally known as the “Active Donor Registration” or ADR system) [1], it has proven highly controversial and has gained minimal support in Dutch parliament. Similar small margins are revealed by studies asking the general public to explicitly evaluate different registration systems [2–6].

This paper argues that in public and political debates on legal donor registration systems attitudes toward organ donation tend to be confounded with the sociopolitical viewpoints that tend to be associated with donor registration systems, causing people to talk past each other and to experience difficulties forming an opinion about these systems. Specifically, those strongly in favor of donation (often represented by patient organizations and health professionals) tend to emphasize that in opt-in countries patients waiting for an organ unnecessarily die, children in particular would be helped enormously by receiving an organ from diseased persons, and an opt-out system is far more effective in reducing patients’ needs. To support the latter argument, they often point to international comparisons showing a much higher number of donors or successful transplantations in opt-out than opt-in countries [7, 8] or to simulation studies conducted in opt-in countries [9, 10]. Yet, they often fail to make explicit that a choice for an opt-out system is also associated with a choice for a more coercive role of the state in obtaining organs for transplantation, and correspondingly with less autonomy [1, 11–13]. In contrast, those objecting against an opt-out system often exclusively refer to its sociopolitical aspects, emphasizing that it violates autonomy and freedom of choice. Their opposition to a change in registration system may give the impression that they are also less in favor of helping needy others and donating organs in particular, although, of course, the one does not follow from the other.

The goal of the present research is to examine how attitudes toward donation interact with the sociopolitical viewpoints that may be associated with donor registration systems and the salience of patients’ need in determining support for a particular registration system. The results will be relevant not only for opt-in countries that still have to arrive at a democratic decision with respect to changing the law to an opt-out system such as the UK but also for a country such as the Netherlands that currently has to decide on how best to obtain the public’s support for a law that was voted for with such a narrow margin. Before presenting the research, the next section will describe how different registration systems tend to be inspired by different sociopolitical perspectives. In two subsequent sections, the nature of attitudes toward organ donation is described, as well as their hypothesized interaction with sociopolitical viewpoints.

## Different Donor Registration Systems Tend to Be Inspired by Different Sociopolitical Perspectives

It has been argued that an opt-in system is associated with respect for individual autonomy according to which “individual moral agents have virtually absolute authority over what happens to their bodies” (14, 38, p.). Although one may argue that *not* taking organs without consent in an opt-in system sometimes also goes against the wishes of the deceased, “people have a right not to have their organs taken but no right to have their organs taken” (13, p. 11).

In contrast, an opt-out system has been associated with violating autonomy [15, 16] and perceived coercion, paving the way for a policy, according to which “the state or government assumes full rights and ownership of an individual’s body and organs at the end of life” (11, p. 29). Furthermore, as Bruce and Koch (15, p. 3262) explain, a lack of objection in the context of an opt-out system “does not justify presuming consent, in the same way that ‘not saying no’ does not equate to saying ‘yes’.” The sociopolitical differences between an opt-in and opt-out system (the first being associated with autonomy, the latter with a coercive role of the state) are not merely theoretical or philosophical but are also perceived as such by the general public. For example, McKenzie et al. [17] found that a majority of their research participants believed that policymakers advocating an opt-out system think that people ought to be donors, whereas a majority believed that an opt-in system does not tell anything about the normative expectations of policymakers.

It should be noted that existing registration systems normally are described in relatively neutral terms and that the underlying sociopolitical viewpoints are relatively hidden. By making explicit the sociopolitical distinctions as clearly as possible, however, the present research will be able to address questions such as: Are those strongly in favor of donation also in favor of coercion and willing to give up freedom of choice? Do those strongly objecting against a coercive role of the state in procuring organs also object to organ donation itself, and vice versa? And how about those still in doubt about their decision whether to become a (non)donor?

In addition to emphasizing autonomy versus coercion, organ donation may also be framed in terms of reciprocity, arguing that in order to realize a fair distribution of a limited good such as posthumously donated organs, people should become aware of their mutual responsibility in supplying enough organs and in avoiding free riding (i.e., taking but not giving organs) [18–20]. It may be argued that an emphasis on reciprocity and social dependency may induce an obligation to donate organs while at the same time reducing a registration system’s association with a coercive role of the state. If so, then reciprocity may become more acceptable than coercion, perhaps also for those who strongly object to donation itself.

## Attitudes Toward Posthumous Organ Donation

Behavioral evidence that people are actually willing to donate or not donate their organs (e.g., carrying a donor codicil or being registered as explicitly confirming that one is a donor or nondonor) seems an especially valid measure of people’s attitude toward donation and its emotional components. In particular, organ donors express more sympathy for patients [21] and less anxiety [22] than nondonors. Relatedly, donors have stronger beliefs about the benefits of organ donation to patients, whereas nondonors have stronger beliefs about different threats associated with thinking about death, unreliability of doctors, or the postmortem treatment of the body (for reviews, see 23, 24–27). Social-psychological research on prosocial and helping behavior provides ample evidence that sympathy motivates people to care for vulnerable and needy others and results in satisfaction after having been able to help successfully [28, 29]. In contrast, as anxiety is associated with the self-preservational goal to establish safe conditions, it negatively affects helping tendencies [30].

## Hypothesized Interactions Between Sociopolitical Perspectives and Attitudes Toward Donation

Support for a particular registration system may be influenced by the extent to which the system’s underlying sociopolitical viewpoint is congruent with the motivational aspects of sympathy and anxiety. In particular, registered donors (probably high in sympathy and low in anxiety; see above) already have made a choice that is in agreement with their motivation to help needy others. They are therefore likely to support a system explicitly based on autonomy because it is associated with an intrinsic motivation to act in altruistic ways [31, 32], as well as a system that seems especially effective in reducing the organ shortage and relieve the needs of patients such as one based on coercion. In contrast, registered nondonors (probably high in anxiety and low in sympathy) already have made a choice that appears safe to them and may therefore respond negatively to a system in which they would be automatically considered donors. Complementarily, an emphasis on coercion and lack of decisional freedom may cause anger and anxiety which negatively affect attitudes toward organ donation itself.

It is less clear how unregistered individuals will respond to different sociopolitical viewpoints, as they are less homogeneous with respect to underlying motivation and emotions. Those who strongly intend to register as a donor or nondonor may respond in similar ways to different viewpoints as registered donors or nondonors, respectively. However, those who are undecided and find it difficult to make a choice may favor a system based on autonomy and that respects their freedom to arrive at a decision when they are ready for it. Yet, they may be especially sensitive to information about the needs of patients waiting for an organ, causing them to react more sympathetically to organ donation. Such a context effect is suggested by research on wording and question-order effects in opinion and attitude surveys, demonstrating that these effects are especially pronounced in individuals who are undecided and ambivalent with respect to the particular issue studied [33].

Although one may expect that a registration system based on reciprocity would be more acceptable than one based on coercion by the state (perhaps especially so among those against donation or still in doubt), it is less clear how it compares to a system entirely based on individual choice and autonomy.

## The Present Research

We conducted two studies to test the hypothesized interaction between sociopolitical viewpoints associated with different registration systems and attitudes toward donation. In study 1, we used current registration status as a proxy for existing attitudes toward donation, and randomly assigned registered donors, registered nondonors, and nonregistered individuals to one of three sociopolitically inspired solutions to reducing the organ shortage, distinguishing between a system based on autonomy, coercion by the state, and reciprocity, respectively. In addition, salience of patients’ need was manipulated as a third independent variable, especially to examine if this variable moderated support for a system based on coercion. In study 2, we concentrated specifically on young and unregistered people in order to find out how prior donation intentions or indecision with respect to donor registration affect their responses to the three different sociopolitical viewpoints. In addition, this study also attempted to manipulate the need of patients in a stronger way than in study 1.

## Study 1

### Method

#### Participants and Design

Participants were three groups of individuals selected from a Dutch internet panel (439 registered donors, 389 registered nondonors, 407 unregistered individuals) who were randomly assigned to a 3 (sociopolitical viewpoint) × 2 (patients’ need—low versus high) experimental design (mean age = 45 years, SD = 14; 53.2% males). Informed consent was obtained from all individual participants included in the study.

This study was carried out in 2013 by a company specialized in online internet research having available a panel of about 16,000 people similar to the Dutch population in demographic characteristics. Members of the panel are reminded that they participate voluntarily and can withdraw from a survey. For a completed survey, they receive a certain number of points which can be exchanged for a gift voucher. The goal was to randomly draw a sample from this panel that would consist of three equally large groups of registered organ donors, registered nondonors, and unregistered persons, and to randomly assign a sufficient number of members of each group to a 3 (socio-political viewpoint) × 2 (patients’ need) experimental design, with about 60 participants in each of its cells (according to prior power considerations, a sufficient number to detect relatively small effects). Based on the relatively small number of registered nondonors in the Dutch population (9.4% vs. 23.4% registered donors and 66% unregistered individuals; Donorregister 2013), and an expected response rate of about 65%, a random sample of 5910 panel members between 18 and 70 years of age was drawn (stratified in terms of age, sex, education, and geographic region) of which 5874 members could be reached by email. From these, 4085 individuals (response rate = 69.5%) answered the question used for selecting the appropriate panel members for the present study. This group did not differ significantly from the nonresponders in terms of age, sex, and education.

From each of the three registration groups, a random sample was drawn of which the members, after logging on to the internet site containing the experimental materials and questionnaires, were randomly assigned to the experimental conditions. Of the 464 selected registered nondonors and the 517 selected donors, 398 (response rate = 85.8%) and 439 individuals (response rate = 84.5%), respectively, logged on to the site and completed the study, whereas this was true for a somewhat smaller number of the 525 selected unregistered individuals (412 of them logged on to the site and completed the study; response rate = 78.5%). Indeed, the significant relation between registration status and participation, *χ*^2^ (2, *N* = 1506) = 11.45, *p* < 0.05, reveals some selection bias. The relatively greater reluctance of the unregistered individuals to answer questions and think about organ donation seems understandable in light of their failure to register their choice with respect to organ donation. Note, however, that drop-out during the study (69 respondents had to be excluded due to low response quality or incompletely filling out questionnaires) for the three registration groups was equally distributed across the six experimental conditions, *χ*^2^ (10, *N* = 1249) = 0.81, *p* = 1.00. For the registered nondonors, registered donors, and unregistered individuals, these six experimental conditions contained 65–67, 69–76, and 65–72 participants, respectively. *χ*^2^ tests indicated that these conditions were equivalent with respect to sex, age, education, religion, and geographic area (all *p*’s > 0.19).

Although we did not specifically ask respondents to indicate if they considered themselves suitable as organ donors, answers to an open-ended question at the end of the questionnaire allowed us to identify and remove 14 respondents who spontaneously considered themselves unsuitable (mostly because of health-related or age-related reasons).

### Procedure

After briefly introducing the issue of postmortal organ donation, the question used to select participants for the main study asked panel members if they already had registered their choice with respect to organ donation by filling out a registration form, and if so, which choice they made. Respondents received general information about the shortage of organs and the fact that different legislative solutions are currently considered. Sociopolitical viewpoint and patients’ needs were manipulated by presenting participants with one of six different texts which they were required to read attentively before continuing to the questionnaire. Once they started to answer the questions, it was impossible for participants to return to the text or to previous questions.

*Socio-political viewpoint* was manipulated at three different levels: autonomy, coercion, and reciprocity. After mentioning that “to ensure that more people register as an organ donor, the following principles should play an important role,” the autonomy text read:

“In a society, freedom and autonomy (the right to determine what happens to our own body) are highly valued. We should be free to make our own decisions and dislike it when those in power tell us what to do, especially so when our own body is concerned. This means that coercion should not play a role in making a decision with respect to organ donation. If we want to be an organ donor after we have died, then so be it. If we don’t want to, then we will not be an organ donor.”

The coercion text read:

“In a society, there is always a certain need for authority and obedience. Without leadership, life would be chaotic, especially in a complex society as ours. Therefore, it is important that the government makes and maintains laws to ensure benefits to society as a whole (think, for example, of paying taxes). This is also true for organ donation. It seems reasonable that the government coerces people to donate their organs after death, by treating everyone automatically as a (registered) donor, without needing their explicit permission. This means, that after you have died, it is allowed to take out your organs and transplant them to a patient currently in need. It will be possible to object to this during your life, but for this, you have to take action yourself.”

The reciprocity text read:

“In a society, people depend on each other. It only makes sense to help and cooperate with others if they are also willing to help you in return. Otherwise, some people have to do all the work while others only reap the benefits. If people are aware of their mutual dependency, cooperation will be fruitful and will have advantages for everyone. However, for this to happen, everyone should stick to rules such as ‘Do to others as you want others to do to you.’ If people follow these principles and register as a donor, then everyone has the same chance of getting an organ when in need.”

Observe that in contrast to other studies examining attitudes toward a reciprocity-based system [20], we refrained from associating reciprocity with prioritization and punishment of free riders.

*Patients’ need* was manipulated by stating in the high need condition that organ donation would relieve the needs of many people, whether young or old, and would give them a better and happier life. In the low need condition, this text was deleted and replaced by a text explaining the technical aspects of postmortal organ transplantation such as favorable conditions (e.g., traffic accidents), brain death, and supplying suitable organs with sufficient oxygen after death.

After filling out the questionnaire, participants were encouraged to consult the complete information about donor registration in the Netherlands as provided by the government.

### Measures

#### Manipulation Checks

At the end of the questionnaire, participants were asked to what extent the text they read emphasized coercion by the government, freedom to decide, mutual responsibility of (and cooperation between) civilians, and the needs of patients (1 = *completely disagree*, 5 = *completely agree*). After appropriate recoding, the first two items (*r* = − 0.56) were combined into a single scale expressing freedom of choice.

*Support for the solution to the organ shortage* was measured with the following three questions: “How acceptable do you find the proposed solution for increasing the number of organ donors?” (1 = *totally unacceptable*, 7 = *totally acceptable*), “If the proposed solution would be implemented, to what extent would you support it?” (1 = *not at all*, 7 = *very much*), and “To what extent does the proposed solution agree with your own viewpoints, norms, and values?” (1 = *does not agree at all*, 7 = *completely agrees*). Cronbach’s alpha for this scale was 0.87.

*Perceived effectiveness of solution* was measured by asking “How effective does the proposed solution seem to you” (1 = *not effective at all*, 7 = *very effective*).

*Sympathy* with patients was measured with the items “Feeling sympathy with patients in need of organs” (1 = *not at all*, 7 = *very much*), “Feeling concern with patients in need of organs” (1 = *not at all*, 7 = *very much*), “If the proposed solution would be implemented, I will be able to save lives by donating my organs” (1 = *very unlikely*, 7 = *very likely*), and “If the proposed solution would be implemented, donating my organs will make me feel good” (1 = *very unlikely*, 7 = *very likely*). Cronbach’s alpha for this scale was 0.85. *Anxiety* was measured with the items “Feeling anxiety about thinking about death and how I will be treated after death” (1 = *not at all*, 7 = *very much*), “If the proposed solution would be implemented, doctors will spend less effort to save my life in case I would be registered as organ donor” (1 = *very unlikely*, 7 = *very likely*), and “If the proposed solution would be implemented, I would be forced to think about all kinds of unpleasant things in relation to dying” (1 = *very unlikely*, 7 = *very likely*). Cronbach’s alpha for this scale was 0.66.

### Statistical Analysis

Hypothesized effects on the dependent measures were examined by a series of 3 (registration status) × 3 (sociopolitical viewpoint) × 2 (patients’ needs) analyses of covariance (ANCOVAs), with sex, age, and education as covariates. In addition, post hoc Tukey HSD tests were performed to examine which significant differences between individual means were responsible for the established effects.

### Results

#### Manipulation Checks

The ANCOVAs on the three manipulation checks revealed that sociopolitical viewpoint had main effects on perceived freedom of choice, *F*(2, 1214) = 40.96, *p* < 0.001, partial *η*^2^ = 0.06, and perceived cooperation, *F*(2, 1214) = 7.99, *p* < 0.01, partial *η*^2^ = 0.01. The pattern of means confirms that the manipulation of this variable was successful, with participants associating more freedom of choice with the autonomy text (*M* = 3.41, SD = 1.04) than with the coercion text (*M* = 2.76, SD = 1.15). The mean for the reciprocity text (*M* = 2.93, SD = 1.06) fell between those for the autonomy and coercion texts; it did not differ significantly from the latter according to a post hoc Tukey HSD test. As intended, the reciprocity text was also perceived as distinctively addressing cooperation among people (*M* = 3.57, SD = 1.00), compared to the coercion (*M* = 3.31, SD = 0.94) and autonomy text (*M* = 3.39, SD = 0.87); according to a Tukey HSD test, only the latter two means did not differ significantly.

The absence of a main effect of patients’ need on perceived need, *F*(2, 1214) = 0.20, *p* = 0.67, suggested that we may not have been successful in manipulating this variable.

We also found main effects of registration status on perceived freedom of choice, *F*(2, 1214) = 38.17, *p* < 0.001, partial *η*^2^ = 0.06; perceived cooperation, *F*(2, 1214) = 31.49, *p* < 0.001, partial *η*^2^ = 0.05; and perceived need, *F*(2, 1214) = 29.96, *p* < 0.001, partial *η*^2^ = 0.05. These effects revealed that, independently of the presented text, registered donors were biased to perceive more freedom of choice, reciprocity, and need (*M*s = 3.35, 3.69, and 3.88, respectively) than unregistered individuals (*M*s = 2.99, 3.35, and 3.59, respectively), followed by registered nondonors (*M*s = 2.74, 3.19, and 3.34, respectively). The ANCOVAs on the manipulation checks revealed no interactions between the independent variables.

#### Emotional Aspects of Registration Status

**Fig. 1:**
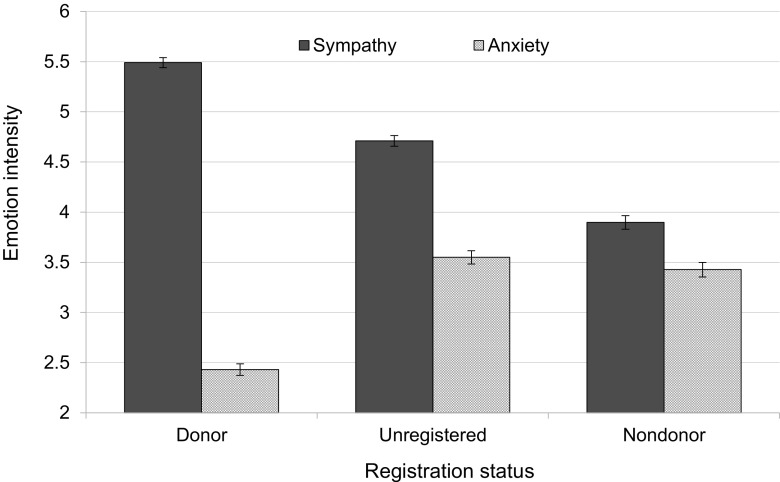
The association between registration status and expressed intensity of sympathy and anxiety (study 1). Standard errors are represented in the figure by error bars attached to each column

Similar ANCOVAs showed that registration status had main effects on anxiety, *F*(2, 1214) = 89.50, *p* < 0.001, partial *η*^2^ = 0.13; and sympathy for patients, *F*(2, 1214) = 199.78, *p* < 0.001, partial *η*^2^ = 0.25. No other significant main effects or interaction effects turned up. As can be seen from Fig. 1, registered donors experienced the highest level of sympathy (*M* = 5.49, SD = 1.04) and the lowest level of anxiety (*M* = 2.43, SD = 1.20), whereas registered nondonors expressed the lowest level of sympathy (*M* = 3.90, SD = 1.32), a level that came close to their level of anxiety (*M* = 3.43, SD = 1.43). Sympathy level of unregistered individuals occupied a middle position (*M* = 4.71, SD = 1.08). Tukey HSD tests showed that the three means for sympathy differed significantly from each other. Furthermore, anxiety of registered donors differed significantly from anxiety of unregistered individuals and nondonors (the latter two did not differ).

#### Support for Sociopolitically Inspired Solutions to the Organ Shortage

**Fig. 2:**
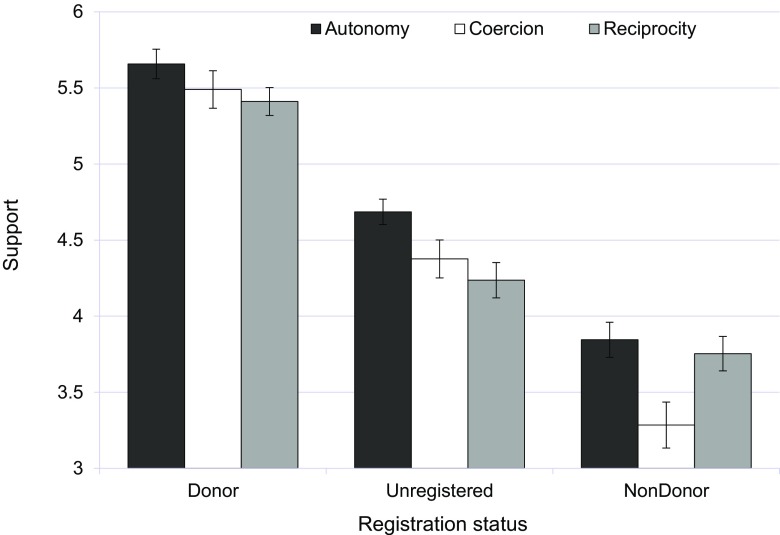
The association between registration status and support for different sociopolitically inspired solutions to the organ shortage (study 1). Standard errors are represented in the figure by error bars attached to each column

A similar ANCOVA on support resulted in main effects of registration status, *F*(2, 1214) = 207.06, *p* < 0.001, partial *η*^2^ = 0.26; and sociopolitical viewpoint, *F*(2, 1214) = 7.49, *p* < 0.05, partial *η*^2^ = 0.01; as well as a marginally significant interaction between these two variables, *F*(4, 1214) = 2.34, *p* = 0.054, partial *η*^2^ = 0.01. As can be seen from Fig. 2, irrespective of sociopolitical viewpoint, registered donors are far more supportive of any solution to the organ shortage than undecided individuals and registered nondonors. Furthermore, the main effect of sociopolitical viewpoint indicates that a solution based on autonomy is generally more valued (*M* = 5.52, SD = 1.27) than one based on reciprocity (*M* = 4.74, SD = 1.28) or coercion by the government (*M* = 3.63, SD = 1.47). No other significant effects turned up.

In order to interpret the interaction between registration status and sociopolitical viewpoint, separate one-way ANOVAs were performed for each of the three registration groups, followed by post hoc Tukey HSD tests. These revealed that sociopolitical viewpoint had no effect on support expressed by registered donors (*p* > 0.24), while having a significant effect for registered nondonors, *F*(2, 386) = 5.54, *p* < 0.01, and unregistered individuals, *F*(2, 404) = 4.55, *p* < 0.01. As shown in Fig. 2, coercion is especially negatively valued by registered nondonors (its mean differs significantly from the means for reciprocity and freedom of choice), whereas autonomy is valued more than both coercion and reciprocity by unregistered individuals (the means of the latter two do not differ significantly).

#### Perceived Effectiveness of Solution to Organ Shortage

A similar ANCOVA on perceived effectiveness yielded main effects of registration status, *F*(2, 1214) = 31.51, *p* < 0.001, partial *η*^2^ = 0.05; and sociopolitical viewpoint, *F*(2, 1214) = 29.85, *p* < 0.001, partial *η*^2^ = 0.05. These variables did not interact significantly. In general, a system based on coercion by the government (*M* = 5.17, SD = 1.52) was seen as more effective than one based on reciprocity (*M* = 4.58, SD = 1.40) or autonomy (*M* = 4.46, SD = 1.41). Furthermore, both registered donors and unregistered individuals (*M* = 5.06, SD = 1.51 and *M* = 4.81, SD = 1.28, respectively) tend to see any solution to the organ shortage as more effective than registered nondonors (*M* = 4.29, SD = 1.53). A Tukey HSD test revealed that only the first two means did not differ.

To summarize, most respondents tend to see coercion as effective in reducing the organ shortage, although only those in favor of donating their organs (and high in sympathy and low in anxiety) are willing to support it. However, while the latter almost equally supported all three sociopolitically inspired solutions, those objecting to donation (and low in sympathy and high in anxiety) showed especially strong rejection of coercion.

## Study 2

In study 1, we were unable to sharply characterize the group of unregistered individuals in terms of their intention to donate or indecision. Within a group of unregistered adolescents, the second study aimed to clearly identify unregistered individuals with different donation intentions and to distinguish them from undecided individuals before exposing them to the experimental conditions.

Second, we have not been successful in manipulating the apparent need state of patients waiting for a donor organ. As an explanation, we propose that our description of high need may have been too abstract to further increase the level of perceived need that is normally associated with the issue of organ donation. A second goal of the next study, therefore, was to manipulate patients’ need in a more salient manner by presenting participants with two concrete patients differing in need for an organ. We hypothesized that a registration system based on coercion will become more acceptable in case the needs of patients are made salient, yet especially for those still in doubt about their donation decisions.

### Method

#### Participants and Design

Participants were 436 pupils from three schools for general secondary education in the Netherlands. Mean age was 16.13 years (SD = 0.90) and 51.1% of the sample comprised boys. They were informed that they would participate voluntarily and could withdraw from the survey at any time. Informed consent was obtained from all individual participants included in the study. Participants were quasi randomly assigned to the same 3 (sociopolitical viewpoint) × 2 (patients’ need) design as in study 1. Because the study was conducted during class, it was not possible to first measure donation intention and then randomly assign participants to experimental conditions. Instead, the six different texts and corresponding questionnaires were randomly distributed in class. After distribution, participants answered a question about their intention and then proceeded to one of the six texts. Because we have no reason to suspect a relation between measured intention and having received a particular text, we believe that this procedure resembles random assignment.

Answers to the question about registration (not filled out by five respondents) revealed that 40 already had registered as a donor. These were excluded from analysis. Of the 391 unregistered participants, 135 intended to register as a donor, 163 indicated that they were still in doubt about their decision, and 54 intended to register as a nondonor. Because we specifically focused on unregistered individuals with a particular donation intention and undecided individuals, the 39 individuals who preferred to leave the decision to others were also excluded from analysis.

#### Procedure and Measures

Sociopolitical viewpoint was manipulated in the same manner as in study 1. In order to manipulate perceptions of patients’ need, participants were presented with a conversation between two peers who talked about their neighbor who was in need for an organ. In the high need condition, the neighbor was described as a basically cheerful 8-year-old girl who felt unhappy due to a failing kidney, frequent hospital visits, and the prospect of a spoiled childhood with less opportunities for contact with peers. In the low need condition, the neighbor was a 50-year-old man who also waited for a new kidney but had learned to cope reasonably well with his condition. Although neither being happy at the moment, he did not suffer from kidney failure during his childhood. Although we realized that this manipulation introduced patient’s age and sex as additional factors, we wanted to make sure that our need manipulation this time would work. It is known that children and females are perceived as more needy than adults and males [34, 35] and as more deserving of help [36].

We used the registration form currently used in the Netherlands to register a choice with respect to posthumous donation, but added the alternative “I would indicate that I am still in doubt about what I will do, and rather would like to wait with filling out the form.” With a few exceptions, the remaining part of the questionnaire was the same as the one used in study 1. Cronbach alphas for sympathy, anxiety, and support were 0.74, 0.72, and 0.84, respectively. Two of the items used to check the manipulation of sociopolitical background appeared to be correlated again, *r* = − 0.59, and therefore combined into a single scale. To examine the effectiveness of the need manipulation, we not only asked about perceived need but also about expected need satisfaction after receiving an organ by asking respondents to what extent the text emphasized that patients would have a better life with a new organ.

### Statistical Analysis

Analyses of covariance (ANCOVA) with registration intention (three levels—donate, in doubt, not donate), sociopolitical viewpoint (three levels—autonomy, coercion, reciprocity), and patients’ need (two levels—low versus high) as independent variables, and with sex and age as covariates, were performed to test the hypothesized effects on the dependent measures, followed by Tukey HSD tests.

### Results

#### Manipulation Checks

The ANCOVAs revealed that sociopolitical viewpoint had main effects on perceived freedom of choice, *F*(2, 331) = 34.46, *p* < 0.001, partial *η*^2^ = 0.17; and perceived cooperation, *F*(2, 331) = 10.31, *p* < 0.001, partial *η*^2^ = 0.06. The pattern of means again confirms that the manipulation of this variable was successful, with participants associating more freedom of choice with the autonomy text (*M* = 4.16, SD = 0.75) than with the coercion text (*M* = 2.85, SD = 1.18), and the mean for the reciprocity text (*M* = 3.13, SD = 1.04) again lying closer to the coercion than autonomy text (a post hoc Tukey HSD test showed that only the latter two means did not differ significantly from each other, *p* = 0.09). Also as intended, the reciprocity text was perceived as distinctively addressing cooperation among people (*M* = 3.84, SD = 0.84), compared to the coercion (*M* = 3.28, SD = 0.92) and autonomy text (*M* = 3.54, SD = 0.91); according to a Tukey HSD test, only the latter two means did not differ significantly (*p* = 0.07).

Although the need manipulation again had no effect on perceived need, *F* < 1, it had an effect on perceived need satisfaction, *F*(2, 331) = 5.21, *p* < 0.05, partial *η*^2^ = 0.02, indicating that the patient in the high need condition was associated with greater need satisfaction (*M* = 4.39, SD = 0.74) than the patient in the low need condition (*M* = 4.17, SD = 0.90).

We also found main effects of donation intention on perceived cooperation, *F*(2, 331) = 6.45, *p* < 0.001, partial *η*^2^ = 0.04; and perceived need, *F*(2, 331) = 9.73, *p* < 0.001, partial *η*^2^ = 0.06. These indicated that individuals intended to register as a donor saw more evidence for patients’ need (*M* = 4.34, SD = 0.74) than individuals still in doubt about their decision (*M* = 4.02, SD = 0.85) or individuals intending not to donate their organs (*M* = 3.85, SD = 1.00). Furthermore, individuals intended to register as a donor saw more evidence for cooperation (*M* = 3.78, SD = 0.91) than the undecided (*M* = 3.41, SD = 0.88) or those intending not to donate their organs (*M* = 3.44, SD = 0.95). Although the main effect on perceived freedom of choice was not significant, it could be observed that individuals not intending to donate saw somewhat less freedom of choice (*M* = 3.24, SD = 1.20) than those intending to donate or still in doubt (*M*s = 3.39, SD = 1.09, and 3.41, SD = 1.20, respectively). The ANCOVAs on the manipulation checks revealed no significant interactions between the independent variables.

#### Emotional Aspects of Registration Status

**Fig. 3:**
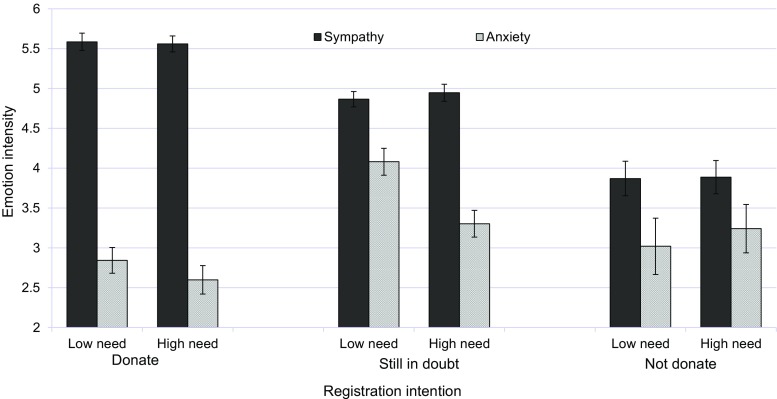
The influence of registration intention and patients’ need on expressed intensity of sympathy and anxiety (study 2). Standard errors are represented in the figure by error bars attached to each column

A similar ANCOVA on sympathy for patients showed that registration intention had a main effect on sympathy, *F*(2, 331) = 68.75, *p* < 0.001, partial *η*^2^ = 0.24. As can be seen from Fig. 3, individuals with donation intention experienced the highest level of sympathy (*M* = 5.57, SD = 0.85), whereas those intended not to donate expressed the lowest level of sympathy (*M* = 3.88, SD = 1.10). Sympathy level of individuals in doubt about their decision occupied a middle position (*M* = 4.90, SD = 0.91). Tukey HSD tests showed that the three means for sympathy differed significantly from each other. No other effects turned up.

The ANCOVA on anxiety revealed a main effect of registration intention, *F*(2, 331) = 14.50, *p* < 0.001, partial *η*^2^ = 0.08, and a marginally significant interaction between registration intention and patients’ need, *F*(2, 331) = 2.56, *p* < 0.07, partial *η*^2^ = 0.01. As can be seen from Fig. 3, individuals with donation intention experienced the lowest, and those still in doubt the highest level of anxiety. However, the latter showed a drop in anxiety when presented with a very needy patient, perhaps making sympathy the more dominant response.

#### Support for the Different Solutions to the Organ Shortage

**Fig. 4:**
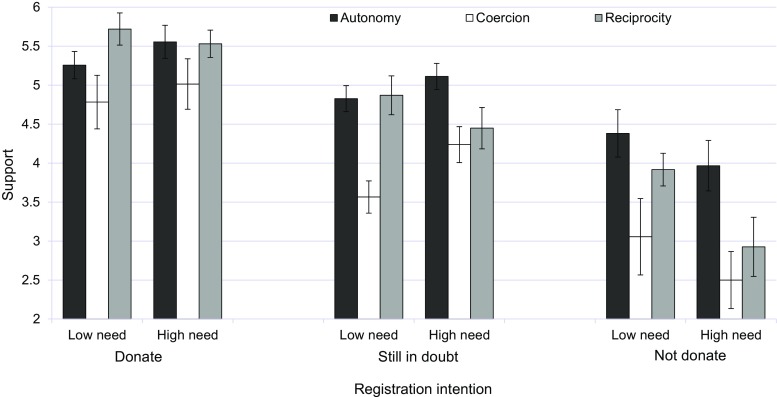
The influence of registration intention and patients’ need on support for different sociopolitically inspired solutions to the organ shortage (study 2). Standard errors are represented in the figure by error bars attached to each column

A similar ANCOVA on support resulted in main effects of registration intention, *F*(2, 332) = 54.42, *p* < 0.001, partial *η*^2^ = 0.24; and sociopolitical viewpoint, *F*(2, 332) = 17.74, *p* < 0.05, partial *η*^2^ = 0.10. As can be seen from Fig. 4, irrespective of socio-political viewpoint, individuals intending to donate are far more supportive of any sociopolitically inspired solution than undecided/uncertain individuals and those intending not to donate. Furthermore, the main effect of sociopolitical viewpoint indicates that a solution based on autonomy is generally more valued (*M* = 5.01, SD = 0.99) than one based on reciprocity (*M* = 4.84, SD = 1.34) or coercion by the government (*M* = 4.07, SD = 1.52). This pattern parallels the pattern obtained for actual donor registrations shown in Fig. 2. Yet the pattern shown in Fig. 4 also suggests that the three independent variables interact. Although the three-way interaction was not significant (*p* > 0.10), and the three two-way interactions appeared to be marginally significant (0.05 < *p* < 0.10), we found it important to explore in greater detail with separate 3 (sociopolitical viewpoint) × 2 (patients’ need) ANOVAs if individuals differing in registrations intentions are differentially sensitive to interactions between sociopolitical viewpoint and the salience of patients’ need.

For individuals intending to donate, the ANOVA only showed a main effect of sociopolitical viewpoint, *F*(2, 127) = 4.48, *p* < 0.05. For those in doubt, the ANOVA resulted not only in a main effect of sociopolitical viewpoint, *F*(2, 155) = 13.32, *p* < 0.001, but also in an interaction of the latter with patients’ need, *F*(2, 155) = 3.13, *p* < 0.05. As can be seen from Fig. 4, only support for a solution to the organ shortage based on coercion was sensitive to the need manipulation, showing an increase in support when going from low to high need. Finally, for intended nondonors, the ANOVA resulted in a main effect of both sociopolitical viewpoint, *F*(2, 46) = 5.45, *p* < 0.01; and patients’ need, *F*(1, 46) = 6.71, *p* < 0.05. Figure 4 shows that these individuals showed reduced support for all three sociopolitical viewpoints when confronted with a patient high in need. The three main effects for sociopolitical viewpoint observed for all three categories of unregistered individuals were due to similar patterns of mean support, with all three categories expressing the least support for coercion (see Fig. 4).

#### Perceived Effectiveness of Solution to the Organ Shortage

A similar ANCOVA on perceived effectiveness yielded main effects of registration intention, *F*(2, 332) = 8.65, *p* < 0.001, partial *η*^2^ = 0.05; and socio-political viewpoint, *F*(2, 332) = 16.93, *p* < 0.001, partial *η*^2^ = 0.09. In general, a system based on coercion by the government (*M* = 5.37, SD = 1.20) was seen as more effective than one based on reciprocity (*M* = 4.68, SD = 1.39) or autonomy (*M* = 4.30, SD = 1.25). Furthermore, both individuals intending to donate and undecided individuals (*M* = 5.04, SD = 1.37 and *M* = 4.79, SD = 1.25, respectively) tend to see any solution to the organ shortage as more effective than those intending not to donate (*M* = 4.09, SD = 1.42). (Tukey HSD test revealed that only the first two means did not differ.)

## Discussion

Sociopolitical viewpoints and attitudes toward organ donation may get mixed up in public and political debates on the desirability of implementing a particular donor registration system. The present research made a first attempt to separate these factors and to show how they contribute in obtaining public support for a particular donor registration system.

It was first confirmed that registration status in study 1 and donation intention in study 2 are associated with emotion profiles representing different attitudes toward organ donation. In particular, registered donors in study 1 and unregistered individuals with donation intention in study 2 showed the highest level of sympathy and the lowest level of anxiety. By comparison, registered nondonors in study 1 and those intending not to donate in study 2 showed the lowest level of sympathy and relatively high levels of anxiety. In agreement with social-psychological research, sympathy and anxiety or distress are known to strongly influence in opposite ways prosocial behavior [28, 30]. Furthermore, irrespective of registration status or donation intention, most participants recognized that a coercive role of the state would result in more effective procurement of organs than a solution based on autonomy or reciprocity.

Differences in emotions seemed to be translated into support for the sociopolitical viewpoint used to argue in favor of a particular solution to the organ shortage. Thus those relatively high in sympathy and low in anxiety and hence most concerned with helping patients (i.e., registered donors in study 1 and unregistered individuals with donation intention in study 2) seemed relatively indifferent with respect to the sociopolitical viewpoint underlying a particular registration system, as long as this viewpoint reflects their current, freely chosen prosocial behavior (i.e., their choice to be a donor in the opt-in system currently in use in the Netherlands), or is recognized as especially effective in obtaining organ donors due to a coercive role assigned to the state. These individuals also showed much stronger support for a coercive role of the state than registered nondonors and those intended to not donate their organs. In contrast, registered nondonors and those intending not to donate (individuals relatively low in sympathy and high in anxiety) strongly distinguished between autonomy and coercion, showing the least support for the latter.

Study 2 also provided evidence that salience of patients’ need may be an additional factor that determines unregistered individuals’ responses to sociopolitically inspired solutions to the organ shortage. In particular, those still in doubt about their decision expressed more support for coercion in response to a patient high than low in need. This effect of need salience was parallelled by a drop in anxiety in response to high need, probably making sympathy and desire to donate a more important determinant of support for a coercive role of the state in solving the organ shortage. An opposite effect of need salience was observed for those intending not to donate their organs who showed decreased support for any solution when the patient was presented as strongly in need for an organ.

The relatively strong effect of need salience on anxiety and support for coercion for those in doubt in study 2 agrees with social-psychological studies showing especially strong effects of context on attitude measurement for those with ambivalent or indifferent attitudes [33]. More to the point, the influence of default options has also been shown to depend on the strength of prior attitudes toward organ donation. In a study performed by van Dalen and Henkes [9] in the Netherlands, participants who reported to be registered as a donor (and presumably strongly positive about donation) were uninfluenced by the hypothetical registration system with which they were presented, with each system generating approximately 90% donors. However, individuals not registered as a donor were considerably influenced by the presented system, with a simulated registration rate of 10% generated by an explicit consent or opt-in system and 37% by a presumed consent or opt-out system. Unfortunately, donation intention of these unregistered individuals was not measured prior to the study. As the present research suggests, intended nondonors and, to a lesser extent, those in doubt about their decision may object to implementing an opt-out system and are less likely to be found among the individuals who say they will register as a donor under an opt-out system.

In the introduction of this paper, we hypothesized that an emphasis on reciprocity may induce an obligation to donate organs while at the same time reducing a registration system’s association with a coercive role of the state. In support of this hypothesis, we found that reciprocity was not only associated with somewhat more freedom of choice than coercion by the state but also was more acceptable than coercion, even for those who strongly objected against donation itself.

Certain limitations of the present studies should be mentioned. First, in order to effectively manipulate differences in sociopolitical viewpoints and to study how these viewpoints interact with prior attitudes toward donation or registration status, we may have artificially suggested or created an unusually strong association between a particular sociopolitical perspective and a particular registration system (i.e., linking autonomy to opt-in and coercion to opt-out). Future research should establish to what extent the particular wording used influenced the present results.^1^

Second, for economical reasons, in study 1 we did not sample the relatively small number of individuals who registered that they would leave it to relatives or friends whether to donate organs, while in study 2 this group was excluded from analysis. There is, however, some reason to believe that these individuals behave in a similar way as undecided individuals. For example, Taels and van Raaij [2] found that many unregistered individuals who first indicated that they could not decide, on a later occasion indicated that they would leave it to family members to decide whether postmortem organ donation could take place. This suggests that at least for some individuals, leaving it to others to decide may be an expression of indecision or ambivalence.

Finally, while study 1 employed a relatively large sample with similar demographic characteristics as the Dutch population, study 2 used a convenience sample of only three schools, making it more difficult to generalize the results.

## Conclusions

We found that individuals most concerned with the needs of patients do not care much about the sociopolitical viewpoints that underlie a particular solution to the organ shortage. They seem to strongly support any system that seems to agree with their own current registration as a donor, or that seems particularly effective in reducing the organ shortage. Yet, the ones who are least in favor of donation itself relatively strongly distinguish between autonomy and coercion, evaluating the latter more negatively. Although one could argue that registered nondonors and unregistered but intended nondonors make up relatively small percentages of the population, their strong sociopolitical objections against an opt-out system may come to dominate the public debate on organ donation, thereby also preventing unregistered and still undecided individuals from considering the social benefits of donating their organs.

For a fruitful and balanced public or political debate about the implementation of a particular donor registration system, we therefore recommend to make the prosocial/donation and sociopolitical dimension equally salient and deserving of debate. In this way, people strongly in favor of donation can be made to recognize that others may not only want to make up their minds about organ donation, but also may want to form a separate opinion about the sociopolitical aspects of different registration systems. Additionally, they may refrain from obscuring the debate by presenting, for example, emotionally charged examples of seriously ill children that are likely to die without a donated organ or who have benefited from receiving one. Complementarily, those tending to focus exclusively on the importance of autonomy and their aversion of coercion by the state may be more willing to consider arguments in favor of helping needy patients waiting for an organ, weighing these against their arguments against donating organs (see also 37).

In closing, it must be remembered that the Dutch Active Donor Registration system to be implemented within the next 2 years represents an unusual variant of an opt-out system that strongly emphasizes freedom of choice and autonomy before presuming consent to posthumous organ donation. Given the right conditions for a balanced and fair debate, it may gain stronger support among the general public than obtained in the Dutch lower and higher house. Perhaps, debate among lawmakers in the UK or particular states in USA that consider to adopt an opt-out system may profit as well from the present considerations, findings, and recommendations.

## Footnotes

1 We performed an additional study with young people in which we presented an opt-in and opt-out registration system, together with a system based on reciprocity and one using obligatory choice, as descriptively and neutrally as possible while also manipulating patients’ need at three different levels. Unfortunately, we were unable to sample enough participants to analyze in a meaningful way the whole study design. However, using the same manipulation checks and comparing only the responses to an opt-in and opt-out system revealed that participants relatively spontaneously associated an opt-out system with a more coercive role for the government (*M* = 3.55, *SD* = 1.66) than an opt-in system (*M* = 2.45, *SD* = 1.27), *t*(127) = 4.23, *p* < 0.001. This suggests that the presently used strong wording may not have artificially introduced a nonexisting sociopolitical basis of the two registration systems.
